# Combination Treatment with EGFR Inhibitor and Doxorubicin Synergistically Inhibits Proliferation of MCF-7 Cells and MDA-MB-231 Triple-Negative Breast Cancer Cells In Vitro

**DOI:** 10.3390/ijms25053066

**Published:** 2024-03-06

**Authors:** Beynon Abrahams, Anthonie Gerber, Donavon Charles Hiss

**Affiliations:** 1 Department of Basic Medical Sciences, Faculty of Health Sciences, University of the Free State, Bloemfontein 9301, South Africa; abrahamsbr@ufs.ac.za (B.A.); 2 Department of Medical Biosciences, Faculty of Natural Sciences, University of the Western Cape, Bellville 7535, South Africa

**Keywords:** breast cancer, triple-negative breast cancer (TNBC), doxorubicin (Dox), epidermal growth factor receptor (EGFR) inhibitor (EGFRi), growth inhibition, drug combination, synergistic interactions, Bliss independence

## Abstract

The role of the epidermal growth factor receptor (EGFR) in tumor progression and survival is often underplayed. Its expression and/or dysregulation is associated with disease advancement and poor patient outcome as well as drug resistance in breast cancer. EGFR is often overexpressed in breast cancer and particularly triple-negative breast cancer (TNBC), which currently lacks molecular targets. We examined the synergistic potential of an EGFR inhibitor (EGFRi) in combination with doxorubicin (Dox) in estrogen-positive (ER+) MCF-7 and MDA-MB-231 TNBC cell lines. The exposure of MDA-MB-231 and MCF-7 to EGFRi produced an IC_50s_ of 6.03 µM and 3.96 µM, respectively. Dox induced MDA-MB-231 (IC_50_ 9.67 µM) and MCF-7 (IC_50_ 1.4 µM) cytotoxicity. Combinations of EGFRi-Dox significantly reduced the IC_50_ in MCF-7 (0.46 µM) and MBA-MB 231 (0.01 µM). Synergistic drug interactions in both cell lines were confirmed using the Bliss independence model. Pro-apoptotic Caspase-3/7 activation occurred in MCF-7 at 0.1–10 µM of EGFRi and Dox single treatments, whilst 1 μM Dox yielded a more potent effect on MDA-MB-231. EGFRi and Dox individually and in combination downregulated the *EGFR* gene expression in MCF-7 and MDA-MB-231 (*p* < 0.001). This study demonstrates EGFRi’s potential for eliciting synergistic interactions with Dox, causing enhanced growth inhibition, apoptosis induction, and downregulation of *EGFR* in both cell lines.

## 1. Introduction

According to global cancer statistics, breast cancer is the most prevalent type of cancer. Although early detection and advanced treatment options have improved survival in high-income countries, mortality remains high in lower-income countries [1]. The majority of breast cancers are hormone receptor-positive (HR+) (85%) and are candidates for endocrine therapies such as tamoxifen and aromatase inhibitors [2,3,4,5,6]. HR+ and HER2+ breast cancers are highly treatable as they possess the biomarkers that serve as molecular targets for therapies, and usually have 5-year cancer-free survival rates of 94–99% [7,8]. However, triple-negative breast cancers (TNBC) are more aggressive and associated with poor prognoses due to the lack of targeted treatments and higher recurrence, with a 5-year breast cancer-free survival rate for stage I TNBC at around 85% [8]. As TNBC tumors lack the hormonal biomarkers for targeted treatment, the systemic therapeutic option that is considered comprises a combination of different classes of chemotherapeutic compounds such as anthracyclines (e.g., doxorubicin) and taxanes [2,9,10,11,12]. Although these strategies work in many cases, the treatment comes with significant side effects that impact the health and quality of life for patients [12]. Furthermore, resistance to several drug treatment regimens is a common development and poses a great concern for health care and effective cancer therapeutics [13]. Over the years, other factors, such as the multidrug resistance transporter (P-glycoprotein) and human epidermal growth factor receptor (EGFR), have also been shown to influence the success of anticancer treatment strategies [14].

Epidermal growth factor receptors (EGFRs) are a subgroup of the protein receptor tyrosine kinase family that consists of four closely related members: EGFR, HER2, HER3 and HER4 [15]. The activation of these receptors is associated with a cascade of events resulting in sustained proliferation and cell survival [15,16]. In particular, EGFR activation following ligand binding potentiates HER-2 function, as HER-2 possesses no known ligands, and instead is activated through other receptor kinases with preferred heterodimerization partners for EGFR [17]. Moreover, the transforming potential and signal pathways that are activated by receptor binding are exclusive. For example, heterodimers EGFR-HER2 are associated with a more vigorous signal cascade [18]. In TNBC (>50%), the altered expressions and mutations of EGFR are common occurrences, which highlight the possible exploitation of EGFR as a molecular target in the development of more effective treatment rationales [15,19,20,21,22].

Currently, several types of targeted therapies for EGFR have been approved for clinical use. EGFR inhibitors include monoclonal antibodies as well as small molecule inhibitors that target the ATP-binding site on the kinase domain [15,23]. For example, one of the most successful FDA approved tyrosine kinase inhibitors (TKIs) indicated clinically for breast cancer treatment is lapatinib, which is used in combination with capecitabine for the treatment of patients with advanced or metastatic breast cancer (MBC) whose tumors overexpress HER2 and who did not respond well to treatment with an anthracycline [24].

The most recently approved TKIs for breast cancer treatment include the following: tucatinib, approved in 2020 for use in combinations with trastuzumab and capecitabine for the treatment of adults with advanced metastatic HER2+ breast cancer; pyrotinib, approved in 2018 in China following a phase III study for use in combination with capecitabine for the treatment of patients with advanced or metastatic HER2+ breast cancer, who were previously treated with anthracycline; neratinib, approved in 2017 for HER2+ breast cancer; and ribociclib, approved in 2017 as a combination therapy with an aromatase inhibitor [25,26,27,28]. Current interest for the effective treatment of TNBC remains in the utilization of TKIs in combination with other classes of compounds [29]. Recent preclinical and clinical successes include a combination of doxorubicin with erlotonib in non-small cell lung cancer and breast cancer as well as the use of synthesized PARP and EGFR inhibitors utilized in combination with adriamycin (doxorubicin) against various cancer cell lines, including MCF-7 and TNBC [30,31]. However, despite this progress, resistance to targeted therapies remains an obstacle [32], thus leading to more intense interest in developing, validating and optimizing combination therapies. Furthermore, TNBC patients remain an underserved population, as targeted therapies for TNBC have been lagging behind. One major impediment to targeted therapies in TNBC is the highly heterogenous nature of this disease, which not only limits efficacy but also results in the rapid emergence of drug resistance, especially under monotherapy regimens [32]. Therefore, combination chemotherapies with targeted therapies have been extensively explored in this context, and clinical trials using this strategy are currently ongoing, including several that are looking specifically at combining EGFR inhibitors with chemotherapeutic drugs [32,33].

To explore multitargeting in the context of combination therapy for the treatment of breast cancer, we investigated the enhanced growth inhibition of an investigational TKI, a 4,6-disubstituted pyrimidine EGFR inhibitor (EGFRi) [34], in combination with doxorubicin (Dox), a DNA damaging anticancer agent, in an ER+ breast cancer cell line, MCF-7, and a TNBC cell line, MDA-MB-231. We observed strong synergistic effects in both breast cancer cell lines and noted that these effects were time-dependent. The results suggest that time dependence is a factor that needs to be carefully documented when examining combination therapies for different classes of anticancer drugs. Significantly, as our main conclusion, this work highlights that combining EGFR inhibitors and chemotherapeutic drugs enhanced the efficacy at cellular and molecular levels in two distinct breast cancer cell lines, which represents an important path towards expanding treatment options for TNBC in particular.

## 2. Results

### 2.1. EGFRi Displays Time-Dependent Increase in Potency When Used as a Single-Agent Whilst Doxorubicin Is More Effective in Combination

**Figure 1:**
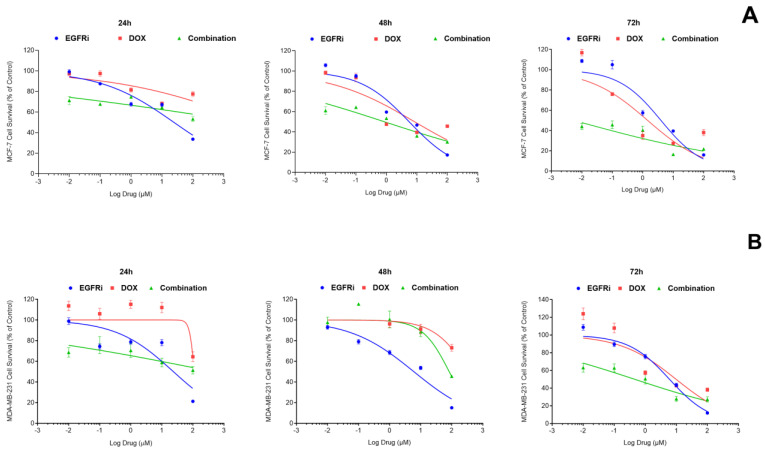
Dose-response curves of the effects of 24, 48 and 72 h exposure of MCF-7 (**A**) and MDA-MB-231 cells (**B**) to EGFRi and Dox, individually and in combination. IC_50_ estimates and corresponding 95% confidence intervals (95% CI) for EGFRi and Dox and combinations were determined via non-linear regression analyses of dose-response data using the variable slope model of GraphPad Prism v10.

To characterize the responses of MCF-7 and MDA-MB-231 cells to EGFRi and Dox as single agents, the dose-response curves were recorded over 72 h (Figure 1A). The dose-response curves exhibited classical hyperbolic or sigmoidal shapes. In MCF-7 cultures, the estimated IC_50s_, i.e., the concentrations that produced half-maximal responses for EGFRi, after 48 h were 5.57 µM (95% CI: 4.146 to 7.478) and after 72 h, 3.96 µM (95% CI: 2.593 to 6.095). In MDA-MB-231 cells, the potency of EGFRi closely resembled that observed in MCF-7 cells with an IC_50_ of 7.05 µM (95% CI: 5.244 to 9.486) observed at 48 h and 6.03 μM (95% CI: 4.566 to 7.965) at 72 h (Figure 1B). Following 72 h of Dox treatments, the IC_50s_ in MCF-7 cells were recorded at 1.40 µM (95% CI: 0.5926 to 3.631) in MCF-7 and 9.67 μM (95% CI: 3.35 to 32.71) in MDA-MB-231 cells. Contextually, the data on single-agent activity provided a baseline for the studies of combination treatment. Furthermore, it highlights the need to examine the time dependence of the effects, as different agents may reach their peak IC_50_ only after a longer time period (as observed for Dox). This, in general, corresponds with previous work that examined the sequential application of EGFR inhibitors and DNA damaging chemotherapy and concluded that the timing of therapy administration is a major factor affecting the outcome [35].

### 2.2. Combination Treatment of EGFRi and Dox Leads to Synergistic Growth Inhibition in MCF-7 and MDA-MB-231 Cells

In addition to single agent treatments, combination treatments with EGFRi and Dox (Figure 1A,B) were performed. At 72 h, the cytotoxicity of the EGFRi-Dox combination was significantly enhanced, with an IC_50_ of 0.01 µM (95% CI: 0.0002703 to 0.027) recorded in MCF-7 cells. For MDA-MB231 cells, the combination effect resulted in an IC_50_ reduction of 0.46 μM (95% (CI: 0.166 to 1.138). Using the data from single-agent dose-response measurements as a benchmark, potency ratios (PRs) were calculated for each of the individual compounds, EGFRi and Dox in relation to the combination of EGFRi-Dox. The PR is a ratio-of-means measure of how more potent the drug is when used in combination than when employed as a single agent. The PRs were determined at 48 h and 72 h treatments to establish the highest potency timeline for subsequent experiments (Table 1). The highest PR of EGFRi (369) in the combination of EGFRi-Dox was recorded following 72 h administration in MCF-7, alluding to the synergistic potential with Dox. This PR for EGFRi in MCF-7 cells was exponentially higher compared to the 48 h treatment timeline (PR:6.55) (Table 1). MDA-MB231 cells were slightly more resilient compared to MCF-7, with a PR of 13.11 recorded for EGFRi at 72 h (Table 1). Dox, a known cytotoxic agent, elicited a PR of 140 in MCF-7 cells at 72 h with a much lower potency in MDA-MB-231 cells (21.02) (Table 1). Both compounds produced their highest individual PRs in the two-drug combination at 72 h, suggesting that the optimal treatment efficacy is achieved at the 72 h time point.

### 2.3. Modeling Synergy Distribution of EGFRi and Dox Combination Treatment in MCF-7 and MDA-MB 231 Cells

**Figure 2:**
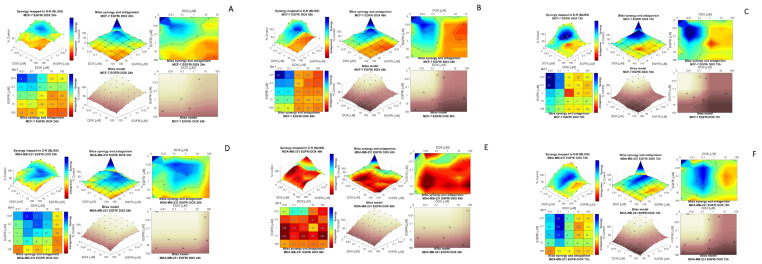
(**A**–**F**): Bliss independence response surface reference model for the dual agent combination effects of 24 h–72 h treatment of MCF-7 (**A**–**C**) and MDA-MB-231 (**D**–**F**) cells with EGFRi and Dox. Each figure (**A**–**F**) is represented as follows—Top panel left: Bliss independence mapping of the synergy levels on the experimental combination dose-response surface | Top Middle pannel: Bliss synergy and antagonism levels visualized as a surface | Top panel right: Contour map of isoboles (iso-effect lines) of Bliss synergy and/or antagonism | Bottom panel left: Bliss model reference of dose-response surface | Bottom panel middle: Bliss synergy and antagonism matrix | Bottom panel right: Bliss model reference of dose-response contour map.

Following the significant dose-dependent reduction in viability together with the significant drug potency ratios of EGFRi-Dox combinations in both cell lines, a drug-drug interaction model analysis for the EGFRi-Dox combination was conducted. Combenefit (Version 2.021) confirmed synergistic interaction between the two compounds, EGFRi and Dox, when used in combination in both MCF-7 and MDA-MB-231 cells. (Figure 2). The results are displayed in a series of 3D dose-response surface plots, synergy and antagonism heat maps or matrices and contour and dose-reduction plots, according to the Bliss independence reference model in a time dependent array. This model was selected for appropriateness as its focus is on two compounds that follow two independent pathways in its mechanism of action [36,37,38,39]. The Loewe additivity model showed similar results The synergy scores can be interpreted as the relative excess response due to drug interactions (i.e., a synergy score of 20 corresponds to 20% of response beyond expectations). Based on unified principles, a synergy score near 0 gives limited confidence on synergy or antagonism, whilst a score of less than −10 indicates that the interaction between two drugs is likely to be antagonistic. A score from −10 to 10 indicates that the interaction between two drugs is likely to be additive, whereas a score larger than 10 is indicative of a synergistic interaction between two drugs. The strength of the interaction is indicated by the colors (blue, strong synergistic; red, strong antagonistic). Overall, the results illustrate synergistic interaction enhancement in a time-dependent manner for both MCF-7 (Figure 2A–C) and MDA-MB-231 (Figure 2C,F) cell lines, with the exception of 48 h, suggesting that at this time, the interaction between the two drugs is likely to be additive. These results are in line with the dose-response combination as depicted in Figure 1 and the lower PR observed in MDA-MB-231 cells at 48 h (Table 1).

In MCF-7 cells, the lowest concentrations of each compound (0.01 µM) at equimolar doses were effective in yielding the highest EGFR-Dox Bliss synergy score of 67 at 72 h (Figure 2C). Similar results were observed when lapatinib, a TKI, and Dox were exposed to MCF-7 breast carcinoma cells [38]. In MDA-MB-231 cells, the highest Bliss synergy score was 44 at 72 h, when EGFRi was used in a 10-fold reduction ratio (1:2) compared to Dox. This further alludes to EGFRi’s ability to potentiate the effect of Dox, in a time-dependent manner (Figure 2F).

### 2.4. Caspase-3/7 Induction in MCF-7 and MDA-MB-231 Cells Following EGFRi and Dox Treatment

**Figure 3:**
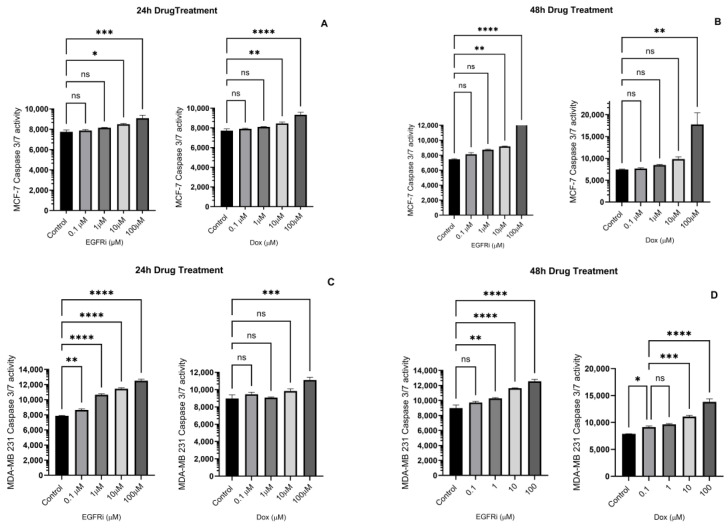
(**A**–**D**): Caspase-3/7 activity following 24 h and 48 h exposure of MCF-7 and MDA-MB-231 cells to EGFRi and Dox. Data were analyzed via one-way ANOVA. All multiple comparisons were performed according to Dunnet’s method, and the overall significance level was set at *p* < 0.05. Significant differences are indicated by (ns = non-significant, * indicates *p* value ≤ 0.05, ** indicates *p* value < 0.01, *** indicates *p* value < 0.001, **** indicates *p* value < 0.0001). Values are means ± SEM (n = 3).

To better understand the changes that led to cell death in MCF-7 and MDA-MB-231 breast carcinoma cells treated with EGFRi and Dox, the cultures were exposed to drugs in a log_10_ dose incremental manner, between 0.1–100 μM over 48 h (Figure 3A–D). Cellular apoptosis was induced by means of cleavage of a four amino acid peptide substrate, Asp-Gly-Val-Asp (DEVD), following activation of Caspases 3 and 7. At 24 h treatment, the EGFRi and Dox induced apoptotic cell killing (*p* < 0.05) at high concentrations of 10 µM and 100 µM in MCF-7 cells (Figure 3A). At 48 h, the same pattern of apoptosis induction was observed in MCF-7 cells for both compounds (Figure 3B). In the MDA-MB231 cell line, the effects of EGFRi were pronounced at 24 h, with apoptosis induction observed at all concentrations, relative to control (Figure 3C). Dox was only effective at a relatively high concentration (100 µM). At 48 h, the cellular killing effect of EGFRi was sustained, whilst Dox also had an effect at 10 µM and 100 µM (Figure 3D). The apoptotic effect of EGFRi corresponds with the cytotoxic effect observed in Figure 1. EGFRi is highly selective for binding EGFR, thus inhibiting the downstream cascade of the PI3K/AKT/mTOR pathway associated with cell survival [34,40].

### 2.5. EGFRi and Dox Synergistically Downregulate the Expression of EGFR Gene

**Figure 4:**
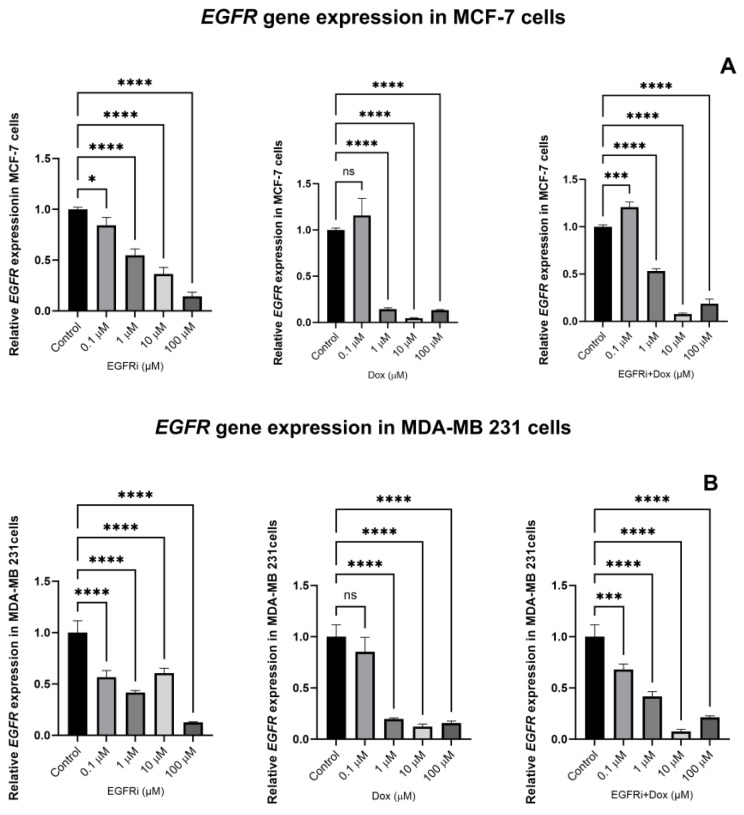
(**A**,**B**): Effects of 48 h pairwise EGFRi and Dox treatments on the expression levels of the *EGFR* gene in MCF-7 (**A**) and MDA-MB-231 TNBC (**B**) breast carcinoma cells. All data are representative of at least three independent experiments and are presented as means ± SEM (n = 3) for cells treated with single agent drugs and combination as indicated. One-way ANOVA analysis was also used to determine significant differences among treatments compared with their respective untreated controls), followed by a Dunnett’s post hoc test to compare all pairs of data sets. Data were considered statistically significant when *p* < 0.05. (ns = non-significant, * indicates *p* value ≤ 0.05, *** indicates *p* value < 0.001, **** indicates *p* value < 0.0001)

To evaluate the extent of EGFRi and Dox inhibition, the expression levels of the *EGFR* gene was evaluated. Total RNA was extracted from both cell lines following 48 h EGFRi and Dox treatment, with an untreated group of cells serving as control. The expression of *EGFR* was significantly downregulated with *p* values dropping below 0.05 following treatment with EGFRi in both MCF-7 and MDA-MB-231 cells (Figure 4A,B). Remarkably, the lowest concentration of EGFRi (0.1 µM) was effective. Dox treatment elicited the same effects of downregulating the expression levels of *EGFR* in MCF-7 and MDA-MB-231 cells. However, the most significant impairment of *EGFR* expression by Dox (*p* < 0.0001) occurred at concentrations of 1 µM and higher. Dox’s ability to dysregulate normal DNA function, one of its many mechanisms of action, is well documented [39,41]. The activation of effector Caspase 3-and 7 directly causes the cleavage of proteins that results in DNA fragmentation, amongst others [42]. The combination treatment of EGFRi-Dox displayed a similar degree of *EGFR* suppression compared to the drugs administered individually in both MCF-7 and MDA-MB-231 cell lines.

## 3. Discussion

The major global challenge in breast cancer continues to be the search for new therapeutic modalities and personalized medicines to cover the enormous spectrum of genetic signatures and hallmarks that present obstacles to breast cancer prevention and eradication [43]. Each cancer subtype presents with its own challenges when it comes to breast cancer therapeutics; for example, in hormone receptor-positive cancers, mutations in molecular drivers have limited the success of treatment efficacy [44]. In TNBC, molecular heterogeneity severely hampers the efficacy of available treatments and is also associated with the development of resistance [2,45]. Furthermore, what further exacerbates treatment failure in patients is the prevailing lack of appropriate molecular targets.

EGFR is the most extensively researched receptor tyrosine kinase due to its role in cancer progression and as a prognostic predicator [46,47,48,49,50,51,52]. As such, EGFR has been a target of interest for anticancer drug discovery and development for several decades, resulting in a range of approved targeted therapies, both TKIs and monoclonal antibodies [53]. Although this receptor tyrosine kinase is also found to be overexpressed in up to 78% of TNBC and correlated with poor overall survival, EGFR-targeted therapies have not shown efficacy when used as monotherapies [32]. In the clinical setting, TNBC requires a multimodal treatment approach involving surgery, radiation and the use of anthracyclines (such as Dox) and taxanes [2,9]. However, DNA-damaging chemotherapy agents, such as Dox, have serious side effects. For example, Dox treatment has been shown to result in senescence and oxidative stress in cardiomyocytes resulting in potentially lethal cardiomyopathy [54]. To overcome such events, many studies have focused on combination therapies with Dox, with the goal of finding synergistic drug combinations that would allow Dox to be used at lower concentrations, thus limiting toxicity, and yet produce the same or improved efficacies.

Furthermore, combination therapies are less likely to lead to drug resistance, which is another important benefit. There have been many preclinical studies on combination treatments with Dox, including a study that examined combining Dox with heat shock protein 90 (Hsp90) inhibitor, gamitrinib, a first-in-class mitochondrial inhibitor that is undergoing clinical trials [55]. The study showed that combination therapy reduced tumor growth in MDA-MB-231 TNBC xenograft models synergistically and without cardiotoxicity. Additionally, Dox was shown to synergize with abemaciclib, a CDK4/6 inhibitor approved for the treatment of breast cancer, in Rb-positive TNBC cells [56]. Another study demonstrated synergistic interactions when lovastatin was used with various chemotherapeutic agents, including Dox [57]. However, other Dox combinations have not performed as well, as illustrated by a recent phase 3 clinical study of Dox in combination with olratumab, an antibody therapy, in metastatic soft tissue sarcoma [58]. Therefore, it is difficult to predict whether a drug combination will be synergistic and to what extent, thus highlighting the need for more empirical data and research in this area. Additionally, Dox resistance is a common occurrence in many cancers. Combinations of different classes of compounds with Dox have been evaluated extensively to attenuate Dox resistance in different cancers [59,60,61,62].

Combination therapy is a central concept in modern medicine, and multi-agent therapies have been a mainstay of cancer treatment [63,64,65,66,67]. When using combination therapy, interactions may be beneficial (additive or synergistic), neutral or harmful (by increasing toxicity and/or decreasing efficacy) [63,68]. Ideally, the two drugs used as a combination therapy synergize to produce a combined activity that is greater than a simple additive effect. An advantage of drug synergy is that it permits therapeutic efficacy to be accomplished with lower doses of individual component interventions, which also reduces probable adverse events [65,68,69]. In this study, we demonstrated that EGFRi and Dox were more effective in inhibiting cell growth in two distinct cell lines, MCF-7 and MDA-MB-231, when combined than when administered individually. The combinatorial use of the two compounds demonstrated the highest level of synergism and significant growth inhibition, as described by the Bliss independence model for drug interactions, at 72 h. We also confirmed that MCF-7 and MDA-MB-231 cells underwent apoptosis by monitoring the Caspase-3/7 markers for apoptosis. We further evaluated the mRNA expression of *EGFR* in EGFRi and Dox-treated and -untreated (control) MCF-7 and MDA-MB-231 cells. Individual EGFRi and Dox as well as combinations of drug treatments demonstrated significant downregulation of the *EGFR* gene in both cell culture systems in a dose-dependent manner. This finding correlates with the level of growth inhibition in our cell cultures.

## 4. Materials and Methods

### 4.1. Cell Lines and Culture Conditions

The cell lines used in this study were acquired from the American Type Tissue Culture Collection (ATCC), following ethics approval (Ethics number 07/3/37) obtained from the Biomedical Research Ethics Committee at the University of the Western Cape, and included an estrogen receptor-positive MCF-7 cell line (ATCC, Rockville, MD, USA Cat No. ATCC HTB-22) and a triple-negative breast cancer MDA-MB-231 cell line (ATCC, Rockville, MD, USA, Cat No. ATCC HTB-26). Frozen 2 mL vials were rapidly thawed and the cells suspended and maintained in pre-heated (37 °C) Dulbecco’s Modified Eagles Medium (DMEM) (Gibco ThermoFisher, Boston, MA, USA, Cat No. 31330038) supplemented with 10% heat-inactivated fetal bovine serum (HIFBS) (Gibco ThermoFisher, Boston, MA, USA, Cat No. 16140071) and 0.2% penicillin/streptomycin (10,000 units/mL penicillin and 10,000 μg/mL streptomycin) (Gibco ThermoFisher, Boston, MA, USA, Cat No. 15140122), grown as monolayer cultures at 37 °C in sterile cell culture flasks and transferred to a CO_2_ air-jacketed incubator (Model NU-5510E NuAire DHD Autoflow (Plymouth, MN, USA)) with 80% relative humidity and 5% CO_2_. The cells were maintained, and once 80% confluency was reached, the cells were trypsinized using 2 mL of 0.25% trypsin-EDTA (Gibco ThermoFisher, Boston, MA, USA, Cat No. 25300120), centrifuged at 5000 rpm, resuspended in a complete medium and dispensed into sterile 96-well culture plates.

### 4.2. Drug Preparation

Doxorubicin hydrochloride (Cat No. 25316-40-9, Sigma-Aldrich, St. Louis, MO, USA), and the EGFR inhibitor (Cat No. 879127-07, Calbiochem, Merck, Darmstadt, Germany) were prepared according to the manufacturer’s instructions. Dox and EGFRi were dissolved in DMSO to ensure biological activity. The compounds were prepared in a log_10_ dose (concentration) range of 0.001, 0.01, 0.1, 1, 10 and 100 μM in a neat medium and administered to cell cultures. The 50% inhibitory concentrations (IC_50s_) of Dox and EGFRi, alone and in combination, on cellular populations were determined as described below.

### 4.3. Cytotoxicity Assay

Both cell lines (MCF-7 and MDA-MB 231) were seeded at a density of 5 × 10^4^ cells/mL into sterile 96-well flat-bottom plates and incubated for 24 h under normal incubation conditions. After cellular adherence (24 h), cell lines were treated with a medium containing increasing log_10_ concentrations (0.001, 0.01, 0.1, 1, 10 and 100 μM) of EGFRi and Dox individually. Cellular cultures were also exposed to Dox and EGFRi in combination at equimolar concentration ratios of 1:1 over the respective time intervals. All experiments were repeated in triplicate. The plates were subsequently incubated for 24, 48 and 72 h. After the incubation period, 20 μL MTT-thiazolyl blue tetrazolium bromide (CAS 298-93-1, Sigma-Aldrich) from a 5 mg/mL MTT stock solution in PBS (Sigma-Aldrich, St. Louis, MO, USA) was added to each well and incubated for 2–4 h. Thereafter, the supernatant was aspirated and 200 μL neat isopropanol added to each well and incubated at room temperature on a vortex shaker for 25 min. Plates were read at 560 nm using the Titertek Multiskan GO model MCC/340 microplate reader.

### 4.4. Caspase-3/7 Assay

MCF-7 and MDA-MB-231 cells were seeded into sterile black 96-well flat bottom plates at a density of 5 × 10^4^ cells/mL and incubated overnight at 37 °C. The cells were exposed to EGFRi and Dox individually at log_10_ concentration ranges 0.1–100 μM for 24 h and 48 h. The Caspase-3/7 reagent in the CellEvent™ Caspase-3/7 assay (Sigma-Aldrich, St. Louis, MO, USA) was prepared, and 100 μL added to the respective wells, after a 100 μL medium containing compounds was aspirated, and incubated at 37 °C for 30 min. Caspase-3 or -7 was activated in apoptotic cells, and the Asp-Gly-Val-Asp (DEVD) peptide substrate was cleaved, which allowed the binding dye to attach to the DNA inside the cells. After incubation, the plates were removed, and the cells imaged using a fluorescent plate reader at absorption and emission wavelengths of 502 nm and 530 nm, respectively.

### 4.5. RT-qPCR–EGFR Gene Expression Analysis

Total RNA was extracted from the treated and untreated MCF-7 and MDA-MB-231 cell cultures using the RNA extraction RNeasy Mini QIAcube Kit (QIAGEN, Hilden, Germany) using the QIAcube instrument according to the manufacturer’s instructions. The extracted RNAs were then quantified using a Nanodrop 8000 Spectrophotometer and the absorbance ratios at 260/280 and 260/230 measured to assure RNA purity. The integrity of each RNA sample was assessed with an RNA 6000 NanoChip kit (Agilent Technologies, Santa Clara, CA, USA) using the Agilent 2100 Electrophoresis Bioanalyzer (Agilent Technologies). Reverse transcription was performed using the Maxima First Strand cDNA Synthesis kit for RT-qPCR with dsDNase (Thermo Scientific, Waltham, MA, USA). Pre-amplification reactions were performed to increase the sensitivity of the samples at low concentrations on an ABI9700 (Applied Biosystems, Waltham, MA, USA) using aliquots of primers for EGFR1 (125 bp), ACTB, GAPDH, HPRT1 and HSPCB, which were pooled to a final concentration of 500 nM each at the following cycling conditions: single cycle at 95 °C for 10 min; 14 cycles at 95 °C for 15 s; 60 °C for 4 min; and single cycle at 99 °C for 10 min.

A qPCR was performed using the QuantStudio 12K Flex Real-Time PCR System (Applied Biosystems) and 2X PowerUp SYBR Green I Mastermix (Thermo Fisher). The primers used for this study were selected from the literature [70,71]. The EGFR primers are as follows: 5′-TCCCTCAGCCACCCA TATGTAC-3′ and 5′-GTCTCGGGCCATTTTGGAGAATTC-3′. The GAPDH primers are as follows: 5′-GAC AGT CAG CCG CAT CTT CT-3′ and 5′-TTA AAA GCA GCC CTG GTG AC-3′. Each reaction was run in triplicate, using the following cycling parameters: UDG activation at 50 °C for 2 min; initial denaturation at 95 °C for 2 min followed by 40 cycles of 95 °C for 15 s, 15 s at the optimized annealing temperature and 72 °C for 1 min. A melt curve analysis was performed on all reactions at the end of the PCR run using default parameters. The amplification data were analyzed with the Life Technologies QuantStudio 12K Flex Software v1.2.4, applying user-defined thresholds to obtain Cq-values, and the expression levels of target genes were compared to housekeeping genes.

### 4.6. Statistical Analysis

Statistical analyses for the cytotoxicity assays were performed via non-linear regression analyses to determine the best-fit IC_50_ estimates and corresponding 95% confidence intervals (95% CI) for EGFRi and Dox, using the [Inhibitor] vs. normalized response-variable slope template from GraphPad Prism (GraphPad Prism version 10.1.2 for Windows, GraphPad Software, San Diego, CA, USA, http://www.graphpad.com, accessed on 14 December 2023). The potency ratio (PR) or dose-reduction index (DRI) for each drug used in equimolar dual combinations with other drugs was computed according to Fieller’s theorem [72], as simplified by Bliss [73]. The drug synergy and combination analysis was performed using the free software program, Combenefit (Version 2.021), which incorporates three drug interaction models—the Loewe additivity model [74,75], the Bliss independence model [73,76] and the highest single agent model [36]. Apoptosis (Caspase-3/7) assays as well as results of the RT-qPCR for EGFR in MCF-7 and MDA-MB-231 breast carcinoma cells were analyzed using a one-way analysis of variance (ANOVA) followed by Dunnett’s multiple comparisons post-hoc test for pairwise analyses between a set of treatments against a single control mean at a significance level of *p* < 0.05. All ANOVA tests were performed using GraphPad Prism for Windows.

## 5. Conclusions

Our findings highlight the potential of using different classes of anticancer compounds in combination in an attempt to enhance efficacy at lower non-toxic concentrations, whilst at the same time providing proof of concept that the synergistic effects of EGFRi with Dox may be a promising therapeutic option and should be evaluated in complex advanced cell-based models, such as 3D-cell models, and in vivo animal studies to evaluate its potential to increase the level of growth inhibition and control cellular differentiation while at the same time reducing toxicity.

## Figures and Tables

**Table 1:** Relative potency ratios (PR) at various time intervals of exposure of MCF-7 and MDA-MB-231 breast carcinoma cell lines to EGFRi + DOX combination.

**MCF-7: Potency Ratios of EGFRi and DOX in EGFRi + DOX Combination**	**Time (h)**	**PR**
Potency Ratio of EGFRi in EGFRi + DOX*	48	6.55
	72	396
Potency Ratio of DOX in EGFRi + DOX	48	9.77
	72	140
**MDA-MB 231: Potency Ratios of EGFRi and DOX in EGFRi + DOX Combination**	**Time (h)**	**PR**
Potency Ratio of EGFRi in EGFRi + DOX*	48	0.29
	72	13.11
Potency Ratio of DOX in EGFRi + DOX	48	4.16
	72	21.02

## Data Availability

The original contributions presented in the study are included in the article, further inquiries can be directed to the corresponding author.
